# Lifestyle Patterns and Incidence of Cardiovascular Diseases, Cancer, Respiratory Diseases, and Type 2 Diabetes: A Large-Scale Prospective Cohort Study

**DOI:** 10.3390/nu17243883

**Published:** 2025-12-12

**Authors:** Qian Zou, Rafael Ogaz-González, Gerton Lunter, Eva Corpeleijn

**Affiliations:** Department of Epidemiology, University Medical Center Groningen, University of Groningen, 9713 AP Groningen, The Netherlands; q.zou@umcg.nl (Q.Z.);

**Keywords:** lifestyle medicine, nutrition, alcohol consumption, non-communicable disease, risk reduction behaviours

## Abstract

**Background**: Lifestyle factors often interact in complex ways when influencing chronic disease risk. We aimed to examine the prospective associations between empirically derived real-life lifestyle patterns (LPs) and the incidence of major chronic diseases, and to explore the linearity of the relationships between lifestyle summation scores and disease risk. **Methods**: We included adults free of cardiovascular diseases (CVDs), cancer, chronic respiratory diseases (CRDs), or type 2 diabetes (T2D) at baseline (2006–2013) from the Dutch Lifelines cohort. LPs and lifestyle summation scores were derived from baseline self-reported data on diet, physical activity, substance use, sleep, stress, and social connectedness, each categorised as healthy, moderately healthy, or unhealthy. Fine–Gray sub-hazard regression models assessed associations between LPs and disease incidence, with natural spline functions used to evaluate linearity in summation scores. **Results**: Among 114,919 T2D-free, 131,248 cancer-free, 91,777 CRD-free, and 77,645 CVD-free participants, we observed 3114 T2D, 4685 cancer, 4133 CRDs, and 2850 CVD incident cases (median follow-up time: 8 years). Compared to the “Unhealthy” pattern, both the “Healthy-in-a-balanced-way” and “Healthy-but-physically-inactive” patterns were broadly significantly protective. The “Unhealthy-but-no-substance-use” pattern was associated with increased T2D risk (Sub-Hazard Ratio (SHR) = 1.27, 95% Confidence Interval (CI): 1.11–1.47) but reduced cancer risk (SHR = 0.85, 95%CI: 0.74–0.97). The “Unhealthy-but-light-drinking-and-never-smoked” pattern was protective for T2D (SHR = 0.89, 95%CI: 0.79–0.99). Linear associations were observed between lifestyle summation scores and disease risk, except for “healthy lifestyle” scores with T2D and “unhealthy lifestyle” scores with CRDs (non-linear *p*-value < 0.05). **Conclusions**: There are potential protective effects of healthy lifestyles on T2D, cancer, CRDs, and CVDs. However, the “Unhealthy but no substance use” demonstrated increased risk on T2D, protective effect on cancer and no significant effect on CRDs or CVDs. The relationship between combined lifestyle factors and NCD risk is complex and partly non-linear, showing diminishing benefits beyond certain thresholds, especially T2D and CRDs.

## 1. Introduction

Non-communicable diseases (NCDs) are the leading causes of death globally [1,2]. Among them, cancer and cardiovascular diseases (CVDs) are the foremost contributors to global mortality [3,4]. Chronic respiratory diseases (CRDs) are the third leading cause of mortality globally in 2019 [5]. Type 2 diabetes (T2D), the most prevalent of diabetes, is predicted to have a 12.2% prevalence rate by 2045 [6]. Collectively, CVDs, cancer, CRDs and T2D account for 80% of all premature NCD deaths [7].

Lifestyle Medicine emphasises prevention and disease management through healthy lifestyle behaviours and avoidance of risky substances defined by six pillars- nutrition, physical (in)activity, sleep, stress, social connection, and substance use [8]. Strong evidence links these lifestyle domains to the incidence of CVDs, cancer, CRDs and T2D [9,10,11]. However, few studies have examined all six pillars simultaneously in relation to these four NCDs [12,13,14,15]. Given the co-occurrence [16,17] and revealed synergistic effect [18,19] of multiple lifestyle factors, it is critical to study their real-world combinations rather than individual effects summed together.

Lifestyle patterns (LPs), identified via latent class analysis (LCA), capture real-world combinations of multiple lifestyle factors [20]. Significant associations have been reported between LPs with cancer [21], T2D [22,23,24], cardiovascular risk markers [25], and dementia [26]. However, comparisons of LP-associated risks for CVDs, cancer, CRDs, and T2D within the same population remain unclear. Lifestyle summation scores are widely used to evaluate combined lifestyle effects [27,28], typically showing that higher scores correlate with lower disease risk [29,30,31]. Yet, few studies have examined whether these associations are linear [29], and comparisons of LPs versus lifestyle summation scores within the same population are lacking.

This study aims to: (1) investigate the relationships between real-world LPs (based on six pillars of Lifestyle Medicine) and four major NCDs within one population, and (2) assess whether associations between lifestyle summation scores and major four NCD risk are linear, as commonly assumed.

## 2. Methods

### 2.1. Study Design and Participants

**Figure 1:**
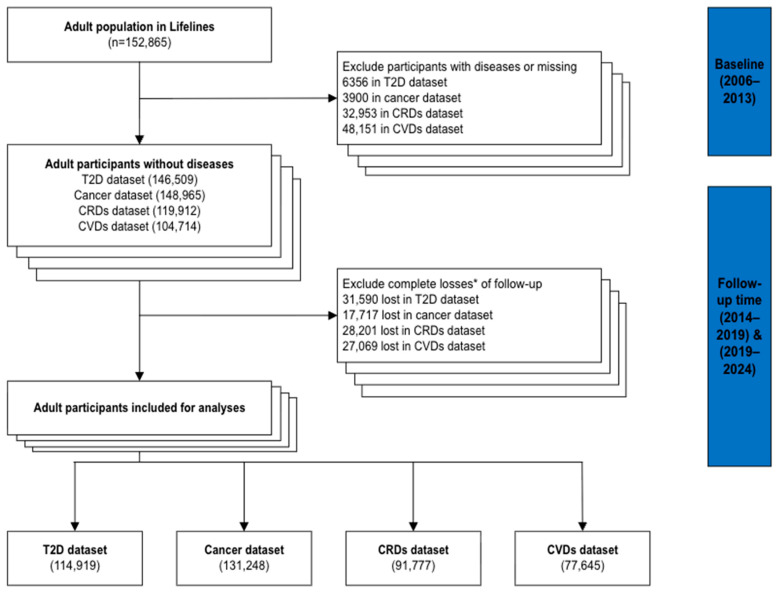
A flowchart of participants exclusion. The stacked boxes represent four separate analysis datasets specified at the bottom of the figure; We first compiled all required variables into a single dataset from the Lifelines cohort and then divided it into four disease-specific analysis datasets, each excluding participants with only one target disease at baseline. To estimate disease incidence, participants were followed from baseline until incident diagnosis or death. * Complete losses of follow-up mean lost to follow-up in wave 2 (2014–2019) and wave 3 (2019–2024).

Data from the Lifelines cohort wave one to wave three were used (2006–2024). Lifelines is a multi-disciplinary prospective population-based cohort study examining in a unique three-generation design the health and health-related behaviours of 167,729 persons living in the North of the Netherlands. It employs a broad range of investigative procedures in assessing the biomedical, socio-demographic, behavioural, physical and psychological factors which contribute to the health and disease of the general population, with a special focus on multi-morbidity and complex genetics [32]. More detailed information about Lifelines has been published elsewhere [33,34]. Adults (≥18 years) who were disease-free at the baseline (wave 1, 2006–2013) were included to meet the research interests. Participants who were lost to follow-up or for whom disease status was unavailable at both follow-up assessments (wave 2, 2014–2019; wave 3, 2019–2024) were excluded (see Figure 1). This study was conducted following the Strengthening the Reporting of Observational Studies in Epidemiology (STROBE) reporting guideline [35].

### 2.2. Exposures and Outcomes

**Figure 2:**
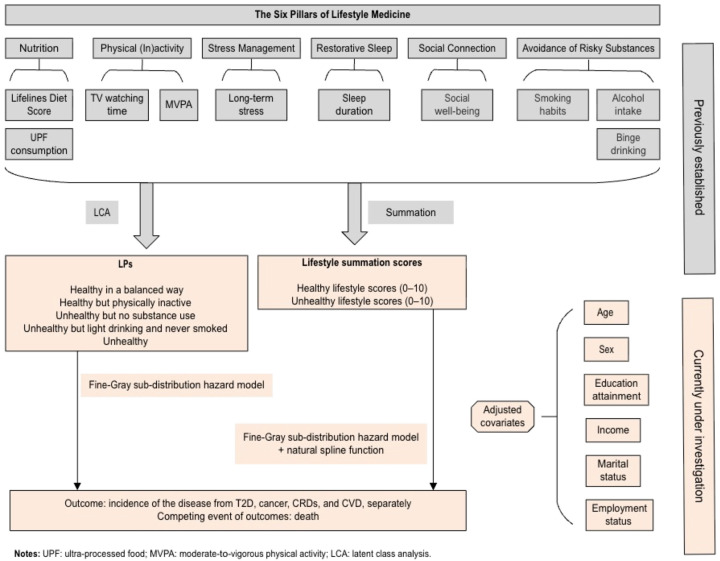
A methodological framework of this study.

LPs and lifestyle summation scores were defined as the primary exposures, derived from baseline data of the Lifelines cohort. Ten lifestyle variables were included based on the six pillars of Lifestyle Medicine [20] and each was categorised into three levels (healthy, moderately healthy, or unhealthy) based on national recommendation guidelines [36], or according to tertiles when no guideline was available: smoking status (never, former, current smoker), alcohol intake (abstainer, light to moderate (0–15 g/day), excessive (≥15 g/day), binge drinking (abstainer, not binge drinking: 1–4 glasses/ day (female) or 1–5 glasses/day (male), binge drinking: ≥4 glasses/day (female) or ≥5 glasses/ day (male)), diet quality (high, moderate, low), ultra-processed food consumption (low, moderate, high), physical activity (>300 mins/week, 120–300 mins/week, 0–120 mins/week), long-term stress level (none, moderate, high), TV watching time (≤2 h, 2–3 h, >3 h), sleep duration (recommend: 7/8/9 h/day (18–65 y) or 7/8 h/day (≥65 y), above recommended: >9 h/day (18–65 y), or >8 h/day (≥65 y), below recommended: <7 h/day (≥18y), and social connections (high, moderate, low). Smoking status and alcohol consumption were determined based on the self-report information. Diet quality was assessed using the Lifelines Diet Score (LLDS) [37], derived from the Food Frequency Questionnaire (FFQ) [38]. Ultra-processed food consumption was classified according to the NOVA framework [39] and expressed as the proportion of UPF weight relative to total weight daily food and beverage consumption, calculated from FFQ responses. Physical activity was quantified using the Short QUestionnaire to ASsess Health-Enhancing Physical Activity (SQUASH) [40], as total weekly minutes of moderate-to-vigorous leisure-time activity, including commuting and leisure. Long-term stress was measured using the Long-term Difficulties Inventory (LDI) [41]. Sedentary behaviour was evaluated by self-reported television watching time. Sleep duration was self-reported in hours and minutes and categorised according to age-specific recommendations of the American National Sleep Foundation [36]. Social connection was evaluated using the short form of the Social Production Function Instrument for the Level of Well-being (SPF-IL(s)) [42]. Further details on the derivation of LPs and lifestyle summation scores are provided elsewhere [20] or Figure 2.

Briefly, we conducted LCA based on the above ten categorical lifestyle variables. The optimal class solution is one that achieved meaningful theoretical interpretation saturation [43,44], rather than relying solely on statistical indices such as Akaike information criterion (AIC), Bayesian information criterion (BIC), Lo–Mendell–Rubin adjusted likelihood ratio test (LMR-LRT), or normalised entropy. Ultimately, a five-class model was selected as the final solution. This process resulted in the identification of two “Healthy” and three “Unhealthy” patterns: “Healthy but physically inactive”, “Healthy in a balanced way”, “Unhealthy but no substance use”, “Unhealthy but light drinking and never smoked”, and “Unhealthy” patterns. Lifestyle summation scores were calculated by summing the number of healthy or unhealthy levels across the ten lifestyle variables separately, resulting in healthy and unhealthy lifestyle scores ranging from 0 to 10.

The outcomes of interest were the incidence of T2D, cancer, CRDs, and CVDs. T2D was defined as self-reported T2D, or self-reported diabetes with missing or unknown type, and/or participants using diabetes medication and/or having a fasting plasma glucose (FG) ≥ 7.0 mmol/L or a hemoglobin A1c (HbA1c) ≥ 6.5% [45]. Individuals with Type 1 diabetes were then excluded, unless they specifically reported T2D and used medications other than insulin, to define pure T2D. Cancer assessment relied on self-reported data; cases involving primary skin cancer, pre-stage cancer, or past cancer with unknown origin were excluded, and considered low prevalence rate and weak associations with the relevant lifestyle factors [46]. CRDs were classified as either asthma or chronic obstructive pulmonary disease (COPD) based on clinical diagnosis, medication use, spirometry, and/or reported two or more symptoms (wheeze, dyspnea at rest, and nocturnal dyspnea) [47]. Eleven CVDs (myocardial infarction, heart failure, atrial fibrillation, heart valve disorders, arrhythmia, aortic aneurysm, stroke, thrombosis, atherosclerosis, narrowing of carotid arteries and balloon angioplasty or bypass surgery) were included based on the International Classification of Diseases (ICD) [48,49]. Mortality data were obtained from the Dutch municipal population registries.

### 2.3. Confounders

Socio-demographic variables were considered as potential confounders, as they are associated with both lifestyle factors and disease outcomes [50,51]. These variables were collected through self-administered questionnaires and included age, sex, marital status, household income, highest educational attainment, and employment status. Household income was normalised by dividing net household income (the midpoint of each participant’s income category) by the square root of the number of household members. The categorisation of these variables is presented in Table 1 and Supplementary Material File S1.

### 2.4. Statistical Analysis

We calculated the incidence rates for T2D, cancer, CRDs and CVDs by dividing new cases by person-years at risk and multiplying by 100,000, respectively. Person-years at risk were calculated from the date of enrolment to the date of death, onset of disease, loss to follow-up, or the end of follow-up, whichever came first. We imputed missing socio-demographic variables and LPs using multivariate imputation by chained equations (MICE) with five imputation sets [52], where LPs were imputed based on their individual lifestyle indicators rather than on the LPs alone. Details on comparisons of pre- and post-imputation datasets are provided in Supplementary Material, File S2, Figure S1 and Table S1.

Fine–Gray sub-distribution hazard models [53] were used to account for the competing risk of death when estimating the incidence of the four leading NCDs in relation to LPs and lifestyle summation scores, separately. Sub-distribution hazard proportionality was test by plotting cumulative incidence curves. All models were adjusted for socio-demographic variables, with age modelled as a continuous variable. Lifestyle summation scores were further modelled using natural spline functions to capture potential non-linear associations. Models were fitted with degree of freedom (*df*) ranging from 2 to 6, and the optimal non-linear models were selected based on the lowest Akaike Information Criterion (AIC). Log-likelihood ratio tests were then performed to compare the linear models (*df* = 1) with the optimal non-linear models, with *p*-values used to assess statistical significance.

Sensitivity analyses were conducted to evaluate the robustness of our findings. First, to reduce the potential bias arising from lifestyle modifications due to other health conditions, we adjusted for the presence of the three other chronic diseases (excluding the disease of interest) in LPs’ model. Second, we redefined reference groups to compare the effects across different LPs. Third, we performed complete-case analyses to evaluate the impact of missing-data handling. Fourth, we applied traditional Cox proportional hazards models without accounting for competing risks. Fifth, to examine the potential interdependence between healthy and unhealthy lifestyle scores, we included the opposite scores as a covariate in each corresponding model.

All analyses were performed using RStudio (R 4.2.2). Multiple imputations were conducted using the mice package [32]; Fine–Gray sub-distribution hazard models were implemented with the tidycmprsk package, and natural splines functions were applied using the splines package. A two-sided *p*-value of less than 0.05 was considered statistically significant through all the analyses. Figure 2 illustrates the methodological framework of this study.

## 3. Results

### 3.1. Study Population and Descriptive Statistics

The final analytical datasets included 114,919 participants without T2D, 131,248 participants for the cancer analysis, 91,777 for CRDs, and 77,645 for CVDs (Figure 1). Participant characteristics are summarised in Table 1. Across all datasets, approximately 60% were female, with a mean age ranging from 45.3 and 46.3 years (SD = 13). Most participants had upper secondary education (38.3–38.7%), an income between €1500 and €1900 (25.4–26.2%), were married or cohabiting (80.2–81.1%), and employed full-time (44.2–46.9%).

Table 2 presents the prevalence of LPs and lifestyle summation scores. The most common LPs were “Healthy in a balanced way” (38.0–40.4%) and “Unhealthy but light drinking and never smoked” (27.9–28.9%). Approximately 80% of participants had 2–5 healthy lifestyle scores, and 70% had 2–4 unhealthy scores.

Over a median follow-up of 9.3 years for T2D, 5.9 years for cancer, 8.5 years for CRDs, and 8.7 years for CVDs, a total of 3114, 4685, 4133, and 2850 disease incidences were recorded, respectively. CRDs had the highest incidence rate (603 per 100,000 person-years), while T2D had the lowest (321 per 100,000 person-years). Cancer and CVDs had incidence rates of 511 and 485 per 100,000 person-years, respectively (Table 1).

### 3.2. Associations Between LPs and Disease Risk

Table 3 summarises the associations between LPs and disease risk. Compared to the “Unhealthy” pattern, the “Healthy in a balanced way” pattern was significantly associated with reduced risks of T2D, cancer, and CRDs. The “Healthy but physically inactive” pattern was linked to significantly lower risks across all four disease outcomes. The “Unhealthy but no substance use” pattern was associated with increased risk of T2D (SHR = 1.27; 95% CI: 1.11–1.47) but decreased cancer risk (SHR = 0.85; 95% CI: 0.74–0.97). The “Unhealthy but light drinking and never smoked” pattern was linked to decreased T2D risk. File S2 Table S2 presents the associations between LPs and disease risk, using different LPs as reference categories.

### 3.3. Linearity of Associations Between Lifestyle Summation Scores and Disease Risk

**Figure 3:**
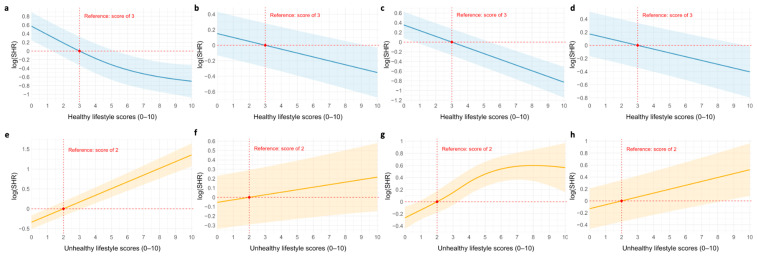
Association curves between lifestyle summation scores and disease risk. Light blue and light orange zones showed corresponding 95% confidence intervals. (**a**,**e**), risk for type 2 diseases; (**b**,**f**) risk for cancer; (**c**,**g**) risk for respiratory diseases; and (**d**,**h**) risk for cardiovascular disease. All models adjusted for age (median), sex (female), marital status (married/cohabiting), income (1500–1900), highest education (upper secondary) and employment status (full-time employed).

Comparison of the optimal non-linear models with the corresponding linear models (Table 4) revealed two associations where the non-linear specification provided a significantly better fit (*p*< 0.05): between healthy lifestyle scores and T2D, and between unhealthy lifestyle scores and CRDs. For the remaining associations, the non-linear models did not demonstrate a significantly improved fit. These associations are illustrated in Figure 3. File S2 Table S3 presents the results of optimal non-linear model selection based on AIC.

Using the most prevalent healthy lifestyle score of 3 as the reference, we observed a significant inverse association with T2D risk (non-linear) and CRD risk (linear) (Figure 3a,c). Only a score of 10 significantly decreased cancer risk (Figure 3b), and no significant associations were found for CVDs (Figure 3d). When using the most common unhealthy lifestyle score of 2 as the reference, unhealthy scores of 0–1 were associated with a significantly reduced T2D risk, while scores of 4–10 were associated with increased risk (Figure 3e). Scores of 4–7 significantly increased CRD risk, with a plateau effect observed at scores 7–10 (Figure 3g). Only a score of 10 significantly increased CVD risk (Figure 3h), and no significant association was found between unhealthy lifestyle scores and cancer risk (Figure 3f).

### 3.4. Sensitivity Analyses

The models we used demonstrated significantly better performance and there was no evidence of violation of proportionality assumption (Table 4, File S2 Table S3, Table S7 and Figure S4). Findings between LPs and disease risk remained generally robust across multiple sensitivity analyses. Results were consistent after additional adjustment for other health conditions (File S2 Table S4), using complete-case analysis—although some SHR became non-significant and the association between the “Unhealthy but no substance use” pattern and CVDs became statistically significant (SHR = 1.19, 95% CI: 1.00–1.42) (File S2 Table S5) and when using Cox proportional hazard models (File S2 Table S6).

When mutually adjusting healthy and unhealthy lifestyle scores for each other, several associations became non-significant (File S2 Figure S2): between healthy lifestyle scores and T2D, healthy lifestyle scores and cancer, and unhealthy lifestyle scores and CVDs. Additionally, associations for lower lifestyle scores (healthy lifestyle scores and CRDs; unhealthy lifestyle scores and T2D) lost statistical significance, and the previously observed plateau effect for CRDs was no longer significant.

## 4. Discussion

In this large prospective Dutch cohort study, we investigated how LPs influence the incidence of four major NCDs and examined the linearity between lifestyle summation scores and disease risk. Our findings demonstrate that while generally two “Healthy” LPs were broadly protective, certain mixed “Unhealthy” patterns exhibited disease-specific associations. We also observed mostly linear associations between lifestyle scores and outcomes, alongside two non-linear associations, suggesting that combined lifestyle–disease relationships may be complex and involve threshold or saturation effects.

### 4.1. Association Between LPs and Disease Risk

Limited research has investigated LPs and the incidence of major NCDs comparatively. Consistent with prior studies, our healthy LPs showed broad protective effects. For instance, a study on LPs and T2D [22] found that both an “unhealthy lifestyle group” and a “poor diet and low physical activity group” were significantly associated with increased T2D risk compared with a “healthy lifestyle group”. Two other studies using principal component analysis (PCA) also linked LPs with disease outcomes [21,23], though results varied. One reported that a pattern characterised by “high socioeconomic status and low physical activity” was associated with elevated serum concentrations of branched-chain amino acids (BCAAs) [23], which increase T2D risk [54]. Our results differed: the “Healthy but physically inactive” pattern was associated with a lower T2D risk. Methodological differences may explain this. LPs used in our study were identified using LCA, a person-oriented approach that identifies subgroups of individuals based on shared characteristics across multiple lifestyle factors. In this context, the “Healthy but physically inactive” pattern reflects individuals with low physical activity levels who nonetheless engage in other healthy lifestyle behaviours (never smoking/drinking alcohol and healthy diet), highlighting the complex interplay of real-life behavioural patterns [20]. In contrast, PCA is a variable-oriented method that reduces dimensionality without necessarily capturing complete individual-level behavioural profiles. Therefore, the “high socioeconomic status and low physical activity” pattern identified by Manninen et al. may reflect limited information on other lifestyle behaviours. As a result, while their findings suggest a detrimental effect, our pattern—encompassing a broader set of healthy behaviours—was protective. Notably, our findings suggest that a healthy diet may partially offset the adverse impact of physical inactivity on T2D risk, highlighting the importance of investigating the combined effects of lifestyle factors. Although prior studies have shown that physical activity can eliminate the increased mortality risk associated with sedentary behaviour [55], it remains unclear whether a healthy diet can similarly compensate for the negative effects of physical inactivity on T2D causally. Our study points to a potential new direction for research in this area.

Whereas our findings demonstrate that generally two “Healthy” LPs were broadly protective, certain mixed “Unhealthy” patterns exhibited disease-specific associations. Disease-specific effects were observed in the “Unhealthy but no substance use” pattern, which was associated with an increased risk of T2D but a decreased risk of cancer. The contrasting effects of alcohol consumption on T2D and cancer risk may partly account for the disease-specific associations we observed. According to a meta-analysis, light to moderate alcohol consumption was associated with a lower risk of T2D, whereas heavy consumption showed little or no additional protective effect [56]. In contrast, alcohol consumption has been associated with an increased risk of cancer in a dose-dependent manner compared to non-consumption [57,58]. This inverse association between light to moderate alcohol consumption and T2D may also explain why the “Unhealthy but light drinking and never smoked” pattern played a protective role for T2D incidence. Notably, no significant associations were observed between the “Unhealthy but no substance use” or “Unhealthy but light drinking and never smoked” patterns and CRDs or CVDs when compared to the “Unhealthy” pattern. However, both patterns were significantly associated with increased risks of CRDs and CVDs when compared with the two “Healthy” LPs (see File S2 Table S2). The primary distinction among the “Unhealthy,” “Unhealthy but no substance use,” and “Unhealthy but light drinking and never smoked” patterns was substance use behaviour. This lack of difference in disease risk suggests that light alcohol consumption or the absence of smoking may not substantially influence the development of CRDs or CVDs in the context of an otherwise unhealthy lifestyle. Conversely, these findings indicate that alcohol consumption may play a particularly influential role in the associations observed with T2D and cancer compared with CRDs and CVDs. Further research is needed to confirm this assumption, which could provide valuable evidence to inform lifestyle intervention guidelines. These findings also suggest that the relationship between combined lifestyle factors and NCD risk is complex. This also raises the question of whether the association between lifestyle summation scores and disease risk are truly linear, especially when all lifestyle factors are equally weighted in these scores.

### 4.2. Linearity Between Lifestyle Summation Scores and Disease Risk

To our knowledge, this is the first Dutch study to examine the linearity between lifestyle summation scores and disease outcomes. One previous Norwegian study found non-linear associations between a healthy lifestyle index and the incidence of lung cancer and postmenopausal breast cancer, but not overall cancer incidence [29]. In our study, we did not observe non-linearity for overall cancer, possibly because grouping all cancers masked subtype-specific effects. Instead, we observed non-linear associations between healthy lifestyle scores and T2D risk, and between unhealthy scores and CRD risk. Both showed plateau effects, suggesting diminishing benefits beyond certain thresholds. Similar ceiling effects were reported in a Korean cohort of 2.4 million participants, where physical activity up to ~1000 MET-min/week reduced T2D prevalence but no further reduction occurred beyond that [59]. Such effects highlight the limitations of assuming linear dose–response relationships and suggest tailoring interventions to account for thresholds.

Several mechanisms may underlie these non-linear associations. First, behavioural mechanism—such as interactions among lifestyle factors—may contribute. Previous research has documented synergistic [18] and additive [60,61] effects, which can complicate risk estimates as behaviours accumulate and may generate non-linear risk patterns. Second, equal weighting in summation scores ignores evidence that lifestyle factors contribute unequally to disease risk [61,62]. For example, T2D risk is strongly influenced by diet, sedentary behaviour, and physical activity, but less by social connectedness [22,63]. In our study, healthier diet, limited screen time, and non-smoking were common at lower lifestyle scores, while social connection and alcohol behaviours dominated at higher scores (see File S2 Figure S3). This imbalance may explain why healthy lifestyle scores showed linear reductions in T2D risk at low–moderate levels but plateaued at higher levels. Third, biological pathways linking lifestyle behaviours to disease are often mediated through biomarkers. For example, one study found that albumin, HDL-C, triglycerides, apolipoprotein A, *C*-reactive protein, and HbA1c collectively explained 23% of the association between overall lifestyle behaviours and microvascular diseases [64]. Other mediators—such as inflammation, dyslipidaemia, and oxidative stress—may also contribute to the observed associations [65].

### 4.3. Insights for Implementation: Comparing LPs and Lifestyle Summation Scores

Addressing multiple behaviours simultaneously has been advocated since the 1970s [66], and evidence supports multiple health behaviour change (MHBC) interventions as more effective than single-behaviour approaches [62,67]. Comparing LPs and lifestyle summation scores offers insights for implementation.

Both LPs and lifestyle summation scores consistently show that healthy behaviours reduce the risk of major NCDs, with summation scores offering a straightforward framework for communication. Notably, we observed non-linear associations: reductions in T2D risk plateaued at higher summation scores, indicating that additional benefits become limited beyond a certain threshold. Nevertheless, promoting all healthy behaviours remains important, as substantial gains can be achieved at low-to-moderate adherence levels. Lifestyle summation scores are particularly useful for individual-level interventions, as they help balance achievable effort with meaningful risk reduction.

Regarding the discrepancies between LPs and lifestyle summation scores, LPs provide more nuanced, disease-specific information. Unlike summation scores, which generally show a monotonic association (more unhealthy behaviours, higher risk), LPs highlighted that certain behaviours within combined lifestyle patterns- such as alcohol consumption- are more prominent in T2D and cancer risk than for CRDs or CVDs. LPs also better capture real-life behavioural complexity and interdependence, making them more suitable for population-level strategies. Previous work showed that LPs correlate with socio-demographic factors [20], enabling identification of high-risk subgroups. Tailored lifestyle interventions that address the contextual and structural barriers of certain LPs could therefore enhance adherence and effectiveness. While the complexity of patterns may pose challenges for practical implementation, trained professionals can manage and translate these insights into actionable strategies.

Integrating insights from both approaches may enable more comprehensive and equitable strategies: summation scores for personalised, simple guidance, and LPs for structural, population-level interventions. Our finding also supports the development of disease-specific lifestyle scores that weigh factors according to their relative impact while considering biomarker effects. Such scores could better reflect nuanced relationships, improve risk prediction, and guide interventions.

### 4.4. Limitations and Strengths

This study has notable strengths. It is a large prospective cohort with a substantial sample size and long follow-up, enhancing statistical power and temporal inference. The use of theory-based domains from the Lifestyle Medicine framework strengthens conceptual clarity of both summation scores and LPs. Studying multiple NCDs in one population enabled cross-disease comparisons. Analyses also accounted for competing risks from death, improving disease-specific estimates.

Several limitations must be acknowledged. Lifestyle behaviours were self-reported and may be prone to recall bias or socially desirable responses. LPs were derived using LCA, which has inherent limitations such as potential class misclassification, sensitivity to model specifications, and dependence on the selected indicators. Although many covariates were adjusted for, residual confounding is possible. Lifestyle factors were measured only at baseline; changes over time were not captured, except partially for smoking. Behavioural changes due to early symptoms may have introduced reverse causation. Missing LPs were present; we imputed missing LPs using observed lifestyle indicators after deriving LPs from complete cases in the LCA. While this approach preserves real-life patterns, it may still introduce some distributional bias compared with imputing lifestyle factors prior to identifying LPs. However, sensitivity analyses showed minimal differences between the pre- and post-imputation datasets, supporting the robustness of our findings despite this limitation. In the sensitivity results, a few associations lost statistical significance. This attenuation likely reflects reduced statistical power in the complete-case datasets and the removal of information gained through imputation. Nonetheless, the direction and magnitude of the estimates generally remained stable, suggesting that the underlying relationships are not fundamentally altered. However, the loss of significance indicates that some associations may be less robust and should be interpreted with appropriate caution. Finally, grouping heterogeneous outcomes (e.g., cancers) into broad categories may obscure subtype-specific effects. Prior work shows lifestyle–disease associations vary across cancer types [57], so future studies should analyse subtypes separately.

## 5. Conclusions

The relationship between combined lifestyle factors and NCD risk is complex and partly non-linear, indicating diminishing benefits beyond certain thresholds, especially T2D and CRDs. While lifestyle summation scores are more straightforward for individual risk communication, LPs capture behavioural complexity and may be more effective for population-level prevention. Findings from both LPs and lifestyle summation scores consistently showed potential protective effects of healthy lifestyles on T2D, cancer, CRDs, and CVDs. However, the “Unhealthy but no substance use” demonstrated increased risk on T2D, protective effect on cancer and no significant effect on CRDs or CVDs, suggesting that the most influential lifestyle factors within combined behaviours may differ across diseases. Overall, these findings underscore the need for integrated, multidimensional lifestyle strategies in chronic disease prevention.

## Figures and Tables

**Table 1:** Baseline characteristics of socio-demographic variables and follow-up information across each dataset.

	T2D Dataset	Cancer Dataset	CRDs Dataset	CVDs Dataset
Sample size	114,919	131,248	91,777	77,645
Disease incidences	3114	4685	4133	2850
Incidence rate ^a^	321	511	603	485
Years of follow-up	9.3 (4.8, 10.8)	5.9 (4.0, 10.0)	8.5 (4.0, 10.3)	8.7 (4.0, 10.3)
Age, year	45.6 ± 12.9	45.4 ± 13.0	46.3 ± 13.0	45.3 ± 12.9
Sex, male	47,570 (41.4)	54,656 (41.6)	38,164 (41.6)	34,024 (43.8)
Education attainment				
Elementary	2799 (2.4)	3622 (2.8)	2245 (2.5)	1842 (2.4)
Lower secondary	29,805 (25.9)	34,719 (26.5)	23,863 (26.0)	19,706 (25.4)
Upper secondary	44,450 (38.7)	50,670 (38.6)	35,097 (38.3)	30,010 (38.7)
Tertiary	35,727 (31.1)	39,720 (30.3)	28,787 (31.4)	24,720 (31.8)
Others	2138 (1.9)	2517 (1.9)	1719 (1.9)	1367 (1.8)
Net income, Euro				
lower than 1100	19,478 (17.0)	22,903 (17.5)	14,842 (16.2)	12,736 (16.4)
1100 to 1500	26,218 (22.8)	29,724 (22.7)	20,828 (22.7)	17,715 (22.8)
1500 to 1900	29,814 (25.9)	33,351 (25.4)	23,952 (26.1)	20,305 (26.2)
Higher than 1900	23,250 (20.2)	26,396 (20.1)	19,191 (20.9)	16,144 (20.8)
I don’t know this	4294 (3.7)	5014 (3.8)	3380 (3.7)	2843 (3.7)
I don’t want to tell	11,865 (10.3)	13,860 (10.6)	9518 (10.4)	7902 (10.2)
Partner relationships				
Married/cohabiting	92,564 (80.6)	105,190 (80.2)	74,416 (81.1)	62,477 (80.5)
Have partner but no-cohabiting	5967 (5.2)	7001 (5.3)	4499 (4.9)	4067 (5.2)
No partner	15,433 (13.4)	17,896 (13.6)	12,062 (13.2)	10,427 (13.4)
Others	955 (0.8)	1161 (0.9)	734 (0.8)	674 (0.9)
Employment status				
Full-time employment	51,516 (44.8)	58,766 (44.8)	40,528 (44.2)	36,408 (46.9)
Retired	10,791 (9.4)	12,663 (9.7)	9646 (10.5)	7125 (9.2)
Housewife/husband	7636 (6.6)	8791 (6.7)	6300 (6.9)	4889 (6.3)
Student	5248 (4.6)	6297 (4.8)	3918 (4.3)	3782 (4.9)
Not employed	6023 (5.2)	6994 (5.3)	4483 (4.9)	3622 (4.7)
Part-time employment	30,201 (26.3)	33,723 (25.7)	23,992 (26.2)	19,546 (25.2)
Less than 12 h/week	3504 (3.1)	4014 (3.1)	2844 (3.1)	2273 (2.9)

Values are mean ± SD, *n* (%) or median (quartile 25%, quartile 75%). ^a^ Unit: 100,000 person-year.

**Table 2:** Disease incidence in each LP and lifestyle summation score across each dataset.

	T2D Dataset		Cancer Dataset		CRDs Dataset		CVDs Dataset	
	New cases/*n*	%	New cases/*n*	%	New cases/*n*	%	New cases/*n*	%
LPs								
Healthy in a balanced way	1074/45,032	39.2	2171/49,855	38	1618/37,051	40.4	1320/30,594	39.4
Healthy but physically inactive	393/13,537	11.8	634/14,529	11.1	523/11,067	12.1	316/8831	11.4
Unhealthy but no substance use	329/8991	7.8	369/11,874	9.1	369/7015	7.7	201/5938	7.7
Unhealthy but light drinking and never smoked	801/32,457	28.2	971/37,692	28.7	1107/25,539	27.9	658/22,455	28.9
Unhealthy	517/14,902	13	540/17,298	13.2	514/11,039	12	355/9827	12.7
Healthy lifestyle scores								
0	56/1182	1	61/1383	1.1	46/847	0.9	35/693	0.9
1	314/8061	7	305/9463	7.2	335/6176	6.7	207/5101	6.6
2	602/19,218	16.7	737/21,940	16.7	759/14,809	16.2	474/12,581	16.2
3	720/26,069	22.7	1024/29,914	22.8	954/20,634	22.5	645/17,537	22.6
4	600/24,555	21.4	1000/27,911	21.3	835/19,668	21.5	586/16,742	21.6
5	395/17,938	15.6	742/20,530	15.6	637/14,793	16.1	448/12,376	15.9
6	253/10,528	9.2	483/11,893	9.1	356/8627	9.4	296/7421	9.6
7	117/5031	4.4	237/5657	4.3	156/4172	4.6	111/3563	4.6
8	45/1814	1.6	71/1989	1.5	44/1527	1.7	38/1240	1.6
9	12/454	0.4	21/500	0.4	<10/395	0.4	<10/341	0.4
10	<10/69	<0.1	<10/68	0.05	<10/63	<0.1	<10/50	<0.1
Unhealthy lifestyle scores								
0	170/9337	8.1	434/10,216	7.8	272/7982	8.7	262/6803	8.8
1	533/21,853	19	1054/24,409	18.6	717/18,347	20	571/15,388	19.8
2	662/27,424	23.9	1190/31,111	23.7	949/22,218	24.2	724/18,602	24
3	640/23,522	20.5	954/26,958	20.5	894/18,645	20.3	560/15,743	20.3
4	517/16,551	14.4	552/19,421	14.8	673/12,756	13.9	377/11,006	14.2
5	293/9339	8.1	298/11,075	8.4	362/6967	7.6	204/5948	7.7
6	193/4563	4	131/5317	4.1	176/3249	3.5	102/2803	3.6
7	77/1717	1.5	53/2018	1.5	74/1154	1.3	33/1007	1.3
8	24/506	0.4	15/592	0.5	13/318	0.4	14/282	0.4
9	<10/102	<0.1	<10/123	<0.1	<10/69	<0.1	<10/60	<0.1
10	<5/5	<0.1	<8/8	<0.1	<6/6	<0.1	<3/3	<0.1

%—the prevalence of each category of LPs/scores within each dataset. To comply with Lifelines’ data use agreements, we refrain from reporting specific case counts for categories with fewer than 10 individuals.

**Table 3:** Associations between LPs and disease risk.

	T2D Incidence	Cancer Incidence	CRDs Incidence	CVDs Incidence
LPs				
Unhealthy	1 (ref)	1 (ref)	1 (ref)	1 (ref)
Healthy in a balanced way	**0.54 (0.49–0.60)**	**0.87 (0.79–0.96)**	**0.79 (0.71–0.88)**	0.91 (0.81–1.03)
Healthy but physically inactive	**0.60 (0.52–0.69)**	**0.82 (0.73–0.93)**	**0.68 (0.60–0.77)**	**0.82 (0.70–0.96)**
Unhealthy but no substance use	**1.27 (1.11–1.47)**	**0.85 (0.74–0.97)**	1.01 (0.88–1.16)	1.16 (0.97–1.38)
Unhealthy but light drinking and never smoked	**0.89 (0.79–0.99)**	0.91 (0.82–1.02)	0.95 (0.86–1.06)	1.05 (0.92–1.20)

All models adjusted for age, sex, education attainment, marital status, income and employment status. It reports sub-hazard ratios (SHR) (95% confidence interval). Bold means the SHR met statistical significance: *p* < 0.05.

**Table 4:** Comparison of linear and optimal non-linear models for the association between lifestyle summation scores and disease risk.

		Degree of Freedom	Log-Likelihood	*χ* ^2^	*df*	*p* Value
Healthy lifestyle scores						
T2D	Linear model	1	−32,499.32	6.40	1	0.011 *
	Optimal non-linear model	2	−32,496.12			
Cancer	Linear model	1	−49,359.52	0	1	0.948
	Optimal non-linear model	2	−49,359.52			
CRDs	Linear model	1	−44,094.37	7.48	3	0.058
	Optimal non-linear model	4	−44,090.63			
CVDs	Linear model	1	−28,666.56	4.51	3	0.211
	Optimal non-linear model	4	−28,664.30			
Unhealthy lifestyle scores						
T2D	Linear model	1	−32,456.71	4.66	3	0.198
	Optimal non-linear model	4	−32,454.38			
Cancer	Linear model	1	−49,372.54	2.44	2	0.295
	Optimal non-linear model	3	−49,371.32			
CRDs	Linear model	1	−44,095.22	7.48	2	0.006 **
	Optimal non-linear model	3	−44,090.06			
CVDs	Linear model	1	−28,665.18	0.63	1	0.428
	Optimal non-linear model	2	−28,664.86			

*df*—degree of freedom. Statistical method was log-likelihood ratio test. * *p* < 0.05; ** *p* < 0.01. Optimal models in this table were selected from five models based on the lowest AIC, see File S2, Table S3.

## Data Availability

Data may be obtained from a third party and are not publicly available. Researchers can apply to use the Lifelines data used in this study. More information about how to request Lifelines data and the conditions of use can be found on their website (https://www.lifelines-biobank.com/researchers/working-with-us (accessed on April 2023 with updating)).

## Supplementary Materials

The following supporting information can be downloaded at: https://www.mdpi.com/article/10.3390/nu17243883/s1, File S1: Definitions of confounders; File S2 Figure S1: Flowchart of exclusion for original datasets; Figure S2: Association curves between lifestyle summation scores and disease risk; Figure S3: Upset plot illustrating the prevalence of different combinations of healthy lifestyle factors; Figure S4: Cumulative incidence curves of diseases by LPs; Table S1: Comparisons between imputed datasets and original datasets; Table S2: Associations between LPs and disease risk using different reference groups; Table S3: AIC values for non-linear models of lifestyle summation scores and disease risk, with degrees of freedom ranging from 2 to 6; Table S4: Associations between LPs and disease risk; Table S5: Associations between LPs and disease risk, complete-case analysis; Table S6: Associations between LPs and disease risk; Table S7: Model Comparison with null models for LPs.

## Abbreviations

CVDs	Cardiovascular diseases
CRDs	Chronic respiratory diseases
T2D	Type 2 diabetes
LPs	Lifestyle patterns
NCDs	Non-communicable diseases
COPD	Chronic obstructive pulmonary disease
LCA	Latent class analysis
FEV1	Forced expiratory volume in one second
FVC	Forced vital capacity
MICE	Multivariate imputation by chained equations
SHR	Sub-hazard ratios
HR	Hazard ratios
CI	Confidence interval
SD	Standard deviation
PCA	Principal component analysis
BACCs	Branched-chain amino acids
MHBC	Multiple health behaviour change
