# Supplementary material for: Lifestyle Patterns and Incidence of Cardiovascular Diseases, Cancer, Respiratory Diseases, and Type 2 Diabetes: A Large-Scale Prospective Cohort Study
Source: Nutrients. 2025 Dec 12;17(24):3883. doi: 10.3390/nu17243883 (PMC12736167; doi:10.3390/nu17243883)
Supplement: Supplementary file 1 [file nutrients-17-03883-s001.zip › Supplementary materials_S2.pdf]

**Supplementary Material File S2: Tables and Figures**

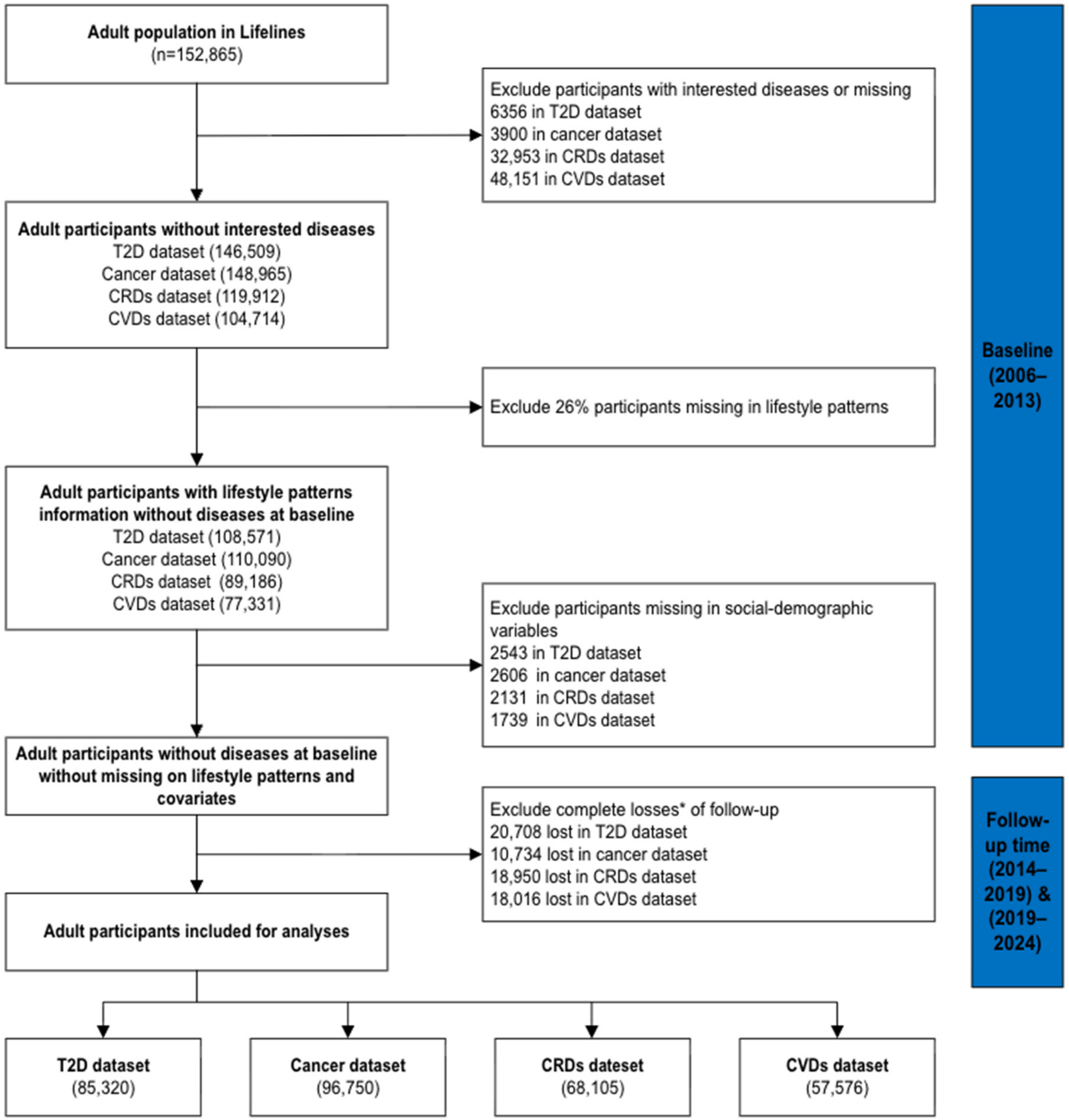


**Figure S1** Flowchart of exclusion for original datasets

*Complete losses of follow-up mean lost to follow-up in wave 2 (2014–2019) and wave 3 (2019–2024).

**Table S1** Comparisons between imputed datasets and original datasets

| | T2D dataset | | Cancer dataset | | CRDs dataset | | CVDs dataset | |
|---|---|---|---|---|---|---|---|---|
| | Imputed dataset | Original dataset | Imputed dataset | Original dataset | Imputed dataset | Original dataset | Imputed dataset | Original dataset |
| **Sample size** | 114,919 | 85,320 | 131,248 | 96,750 | 91,777 | 68,105 | 77,645 | 57,576 |
| **New cases** | 3114 | 2154 | 4685 | 3436 | 4133 | 3015 | 2850 | 1958 |
| **Incidence rate**[a] | 321 | 307 | 511 | 519 | 603 | 608 | 485 | 460 |
| **Follow-up year** | 9.3 (4.8,10.8) | 9.3 (4.7,10.6) | 5.9 (4.0, 10.0) | 5.8 (4.0, 9.9) | 8.5 (4.0, 10.3) | 8.6 (3.9, 10.1) | 8.7 (4.0, 10.3) | 8.8 (4.0, 10.2) |
| **Age, year** | 45.6 ± 12.9 | 45.4 ± 12.5 | 45.4±13.0 | 45.1±12.6 | 46.3±13.0 | 46.1±12.6 | 45.3±12.9 | 45.1±12.5 |
| **Sex, male** | 47,570 (41.4%) | 35,094 (41.1%) | 54,656 (41.6%) | 40,041 (41.4%) | 38,164 (41.6%) | 28,158 (41.3%) | 34,024 (43.8%) | 25,146 (43.7%) |
| **LPs** | | | | | | | | |
| Healthy in a balanced way | 45,032 (39.2%) | 33,224 (38.9%) | 49,855 (38.0%) | 36,801 (38.0%) | 37,051 (40.4%) | 27,374 (40.2%) | 30,594 (39.4%) | 22,378 (38.9%) |
| Healthy but physically inactive | 13,537 (11.8%) | 7025 (8.2%) | 14,529 (11.1%) | 8125 (8.4%) | 11,067 (12.1%) | 5799 (8.5%) | 8831 (11.4%) | 4633 (8.1%) |
| Unhealthy but no substance use | 8991 (7.8%) | 6609 (7.8%) | 11,874 (9.1%) | 7852 (8.1%) | 7015 (7.7%) | 5189 (7.6%) | 5938 (7.7%) | 4407 (7.7%) |
| Unhealthy but light drinking and never smoked | 32,457 (28.2%) | 26,859 (31.5%) | 37,692 (28.7%) | 30,589 (31.6%) | 25,539 (27.9%) | 21,091 (31.0%) | 22,455 (28.9%) | 18,446 (32.0%) |
| Unhealthy | 14,902 (13.0%) | 11,603 (13.6%) | 17,298 (13.2%) | 13,383 (13.8%) | 11,039 (12.0%) | 8652 (12.7%) | 9827 (12.7%) | 7712 (13.4%) |
| **Healthy lifestyle scores** | | | | | | | | |
| 0 | 1182 (1.0%) | 4360 (5.1%) | 1383 (1.1%) | 5083 (5.3%) | 847 (0.9%) | 3322 (4.9%) | 693 (0.9%) | 2678 (4.7%) |
| 1 | 8061 (7.0%) | 13,355 (15.7%) | 9463 (7.2%) | 15,182 (15.7%) | 6176 (6.7%) | 10,297 (15.1%) | 5101 (6.6%) | 8751 (15.2%) |
| 2 | 19,218 (16.7%) | 20,184 (23.7%) | 21,940 (16.7%) | 22,849 (23.6%) | 14,809 (16.2%) | 15,936 (23.4%) | 12,581 (16.2%) | 13,596 (23.6%) |
| 3 | 26,069 (22.7%) | 19,838 (23.3%) | 29,914 (22.8%) | 22,395 (23.2%) | 20,634 (22.5%) | 15,909 (23.4%) | 17,537 (22.6%) | 13,505 (23.5%) |
| 4 | 24,555 (21.4%) | 14,443 (16.9%) | 27,911 (21.3%) | 16,332 (16.9%) | 19,668 (21.5%) | 11,757 (17.3%) | 16,742 (21.6%) | 9872 (17.2%) |
| 5 | 17,938 (15.6%) | 8049 (9.4%) | 20,530 (15.6%) | 9079 (9.4%) | 14,793 (16.1%) | 6620 (9.7%) | 12,376 (15.9%) | 5645 (9.8%) |
| 6 | 10,528 (9.2%) | 3564 (4.2%) | 11,893 (9.1%) | 4077 (4.2%) | 8627 (9.4%) | 2991 (4.4%) | 7421 (9.6%) | 2481 (4.3%) |
| 7 | 5031 (4.4%) | 1170 (1.4%) | 5657 (4.3%) | 1338 (1.4%) | 4172 (4.6%) | 960 (1.4%) | 3563 (4.6%) | 800 (1.4%) |
| 8 | 1814 (1.6%) | 309 (0.4%) | 1989 (1.5%) | 354 (0.4%) | 1527 (1.7%) | 268 (0.4%) | 1240 (1.6%) | 214 (0.4%) |
| 9 | 454 (0.4%) | 45 (0.05%) | 500 (0.4%) | 59 (0.06%) | 395 (0.4%) | 42 (0.06%) | 341 (0.4%) | 31 (0.05%) |
| 10 | 69 (0.06%) | <10 (<=0.01%) | 68 (0.05%) | <10 (<=0.01%) | 63 (0.07%) | <10 (<=0.01%) | 50 (0.06%) | <10 (<=0.01%) |
| **Unhealthy lifestyle scores** | | | | | | | | |
| 0 | 9337 (8.1%) | 7182 (8.4%) | 10,216 (7.8%) | 7939 (8.2%) | 7982 (8.7%) | 6130 (9.0%) | 6803 (8.8%) | 5227 (9.1%) |
| 1 | 21,853 (19.0%) | 16,576 (19.4%) | 24,409 (18.6%) | 18,473 (19.1%) | 18,347 (20.0%) | 13,859 (20.4%) | 15,388 (19.8%) | 11,552 (20.1%) |
| 2 | 27,424 (23.9%) | 20,625 (24.2%) | 31,111 (23.7%) | 23,207 (24.0%) | 22,218 (24.2%) | 16,752 (24.6%) | 18,602 (24.0%) | 13,917 (24.2%) |
| 3 | 23,522 (20.5%) | 17,650 (20.7%) | 26,958 (20.5%) | 20,045 (20.7%) | 18,645 (20.3%) | 13,926 (20.5%) | 15,743 (20.3%) | 11,868 (20.6%) |
| 4 | 16,551 (14.4%) | 12,152 (14.3%) | 19,421 (14.8%) | 14,033 (14.5%) | 12,756 (13.9%) | 9,385 (13.8%) | 11,006 (14.2%) | 8095 (14.1%) |
| 5 | 9339 (8.1%) | 6709 (7.9%) | 11,075 (8.4%) | 7887 (8.2%) | 6967 (7.6%) | 4992 (7.3%) | 5948 (7.7%) | 4301 (7.5%) |
| 6 | 4563 (4.0%) | 3059 (3.6%) | 5317 (4.1%) | 3566 (3.7%) | 3249 (3.5%) | 2170 (3.2%) | 2803 (3.6%) | 1847 (3.2%) |

| | | | | | | | | |
|---|---|---|---|---|---|---|---|---|
| 7 | 1717 (1.5%) | 1057 (1.2%) | 2018 (1.5%) | 1239 (1.3%) | 1154 (1.3%) | 695 (1.0%) | 1007 (1.3%) | 602 (1.1%) |
| 8 | 506 (0.4%) | 263 (0.3%) | 592 (0.5%) | 304 (0.3%) | 318 (0.4%) | 165 (0.2%) | 282 (0.4%) | 144 (0.3%) |
| 9 | 102 (0.09%) | 42 (0.05%) | 123 (0.09%) | 51 (0.05%) | 69 (0.08%) | 28 (0.04%) | 60 (0.08%) | 21 (0.04%) |
| 10 | <10 (<=0.01%) | <10 (<=0.01%) | <10 (<=0.01%) | <10 (<=0.01%) | <10 (<=0.01%) | <10 (<=0.01%) | <10 (<=0.01%) | <10 (<=0.01%) |
| **Education attainment** | | | | | | | | |
| Elementary | 2799 (2.4%) | 1472 (1.7%) | 3622 (2.8%) | 1790 (1.9%) | 2245 (2.5%) | 1148 (1.7%) | 1842 (2.4%) | 912 (1.6%) |
| Lower secondary | 29,805 (25.9%) | 20,753 (24.3%) | 34,719 (26.5%) | 23,834 (24.6%) | 23,863 (26.0%) | 16,598 (24.4%) | 19,706 (25.4%) | 13,646 (23.7%) |
| Upper secondary | 44,450 (38.7%) | 33,250 (39.0%) | 50,670 (38.6%) | 37,899 (39.2%) | 35,097 (38.3%) | 26,288 (38.6%) | 30,010 (38.7%) | 22,436 (39.0%) |
| Tertiary | 35,727 (31.1%) | 28,313 (33.2%) | 39,720 (30.3%) | 31,487 (32.5%) | 28,787 (31.4%) | 22,838 (33.5%) | 24,720 (31.8%) | 19,600 (34.0%) |
| Others | 2138 (1.9%) | 1532 (1.8%) | 2517 (1.9%) | 1740 (1.8%) | 1719 (1.9%) | 1233 (1.8%) | 1367 (1.8%) | 982 (1.7%) |
| **Net income, Euro** | | | | | | | | |
| lower than 1100 | 19,478 (17.0%) | 13,419 (15.7%) | 22,903 (17.5%) | 15,710 (16.2%) | 14,842 (16.2%) | 10,333 (15.2%) | 12,736 (16.4%) | 8846 (15.4%) |
| 1100 to 1500 | 26,218 (22.8%) | 19,169 (22.5%) | 29,724 (22.7%) | 21,759 (22.5%) | 20,828 (22.7%) | 15,260 (22.4%) | 17,715 (22.8%) | 12,974 (22.5%) |
| 1500 to 1900 | 29,814 (25.9%) | 23,337 (27.4%) | 33,351 (25.4%) | 26,003 (26.9%) | 23,952 (26.1%) | 18,661 (27.4%) | 20,305 (26.2%) | 15,815 (27.5%) |
| Higher than 1900 | 23,250 (20.2%) | 18,278 (21.4%) | 26,396 (20.1%) | 20,459 (21.2%) | 19,191 (20.9%) | 15,034 (22.1%) | 16,144 (20.8%) | 12,578 (21.9%) |
| I don't know this | 4294 (3.7%) | 2808 (3.3%) | 5014 (3.8%) | 3157 (3.3%) | 3380 (3.7%) | 2183 (3.2%) | 2843 (3.7%) | 1827 (3.2%) |
| I don't want to tell | 11,865 (10.3%) | 8309 (9.7%) | 13,860 (10.6%) | 9662 (10.0%) | 9518 (10.4%) | 6634 (9.7%) | 7902 (10.2%) | 5536 (9.6%) |
| **Partner relationships** | | | | | | | | |
| Married/cohabiting | 92,564 (80.6%) | 69,128 (81.0%) | 105,190(80.2%) | 78,019 (80.6%) | 74,416 (81.1%) | 55,546 (81.6%) | 62,477 (80.5%) | 46,632 (80.9%) |
| Have partner but no-cohabiting | 5967 (5.2%) | 4438 (5.2%) | 7001 (5.3%) | 5159 (5.3%) | 4499 (4.9%) | 3406 (5.0%) | 4067 (5.2%) | 3000 (5.2%) |
| No partner | 15,433 (13.4%) | 11,119(13.0%) | 17,896 (13.6%) | 12,816 (13.3%) | 12,062 (13.2%) | 8675 (12.7%) | 10,427 (13.4%) | 7526 (13.1%) |
| Others | 955 (0.8%) | 635 (0.7%) | 1161 (0.9%) | 756 (0.8%) | 734 (0.8%) | 478 (0.7%) | 674 (0.9%) | 418 (0.7%) |
| **Employment status** | | | | | | | | |
| Full-time job | 51,516 (44.8%) | 39,064 (45.8%) | 58,766 (44.8%) | 44,515 (46.0%) | 40,528 (44.2%) | 30,779 (45.2%) | 36,408 (46.9%) | 27,710 (48.1%) |
| Retired | 10,791 (9.4%) | 7042 (8.3%) | 12,663 (9.7%) | 7944 (8.2%) | 9646 (10.5%) | 6325 (9.3%) | 7125 (9.2%) | 4555 (7.9%) |
| Housewife/husband | 7636 (6.6%) | 5390 (6.3%) | 8791 (6.7%) | 6094 (6.3%) | 6300 (6.9%) | 4389 (6.4%) | 4889 (6.3%) | 3395 (5.9%) |
| Studying | 5248 (4.6%) | 3946 (4.6%) | 6297 (4.8%) | 4690 (4.9%) | 3918 (4.3%) | 2969 (4.4%) | 3782 (4.9%) | 2817 (4.9%) |
| No jobs | 6023 (5.2%) | 4503 (5.3%) | 6994 (5.3%) | 5201 (5.4%) | 4483 (4.9%) | 3378 (5.0%) | 3622 (4.7%) | 2716 (4.7%) |
| Part-time job | 30,201 (26.3%) | 22,938 (26.9%) | 33,723 (25.7%) | 25,543 (26.4%) | 23,992 (26.2%) | 18,298 (26.9%) | 19,546 (25.2%) | 14,785 (25.7%) |
| Less than 12 h/week | 3504 (3.1%) | 2437 (2.9%) | 4014 (3.1%) | 2763 (2.9%) | 2844 (3.1%) | 1967 (2.9%) | 2273 (2.9%) | 1598 (2.8%) |

Notes: The consequence of the imputation was that more participants in the "Healthy but physically inactive" pattern were included but relatively less from the "Unhealthy but light drinking and never smoked" pattern. Follow-up time, age, gender distribution remained virtually unchanged. Imputation resulted in a better representativeness of (cases from) lower SES groups, since these were more likely to have one or a few covariates missing.

Values are mean ± SD, *n* (%) or median (quartile 25%, quartile 75%)

[a] Unit: 100,000 person-year

To comply with Lifelines data use agreements, we refrain from reporting specific case counts for categories with fewer than 10 individuals.

**Table S2** Associations between LPs and disease risk using different reference groups

| Healthy in a balanced way | Healthy but physically inactive | Unhealthy but no substance use | Unhealthy but light drinking and never smoked | Unhealthy |
|---|---|---|---|---|
| | | **T2D** | | |
| Ref (1.00) | 1.10 (0.98–1.24) | **2.35 (2.07-2.67)** | **1.64 (1.49-1.80)** | **1.85 (1.66-2.05)** |
| 0.91 (0.80–1.02) | Ref (1.00) | **2.13 (1.84–2.47)** | **1.49 (1.31–1.69)** | **1.67 (1.46–1.92)** |
| **0.43 (0.37–0.48)** | **0.47 (0.40–0.54)** | Ref (1.00) | **0.70 (0.61–0.79)** | **0.78 (0.68–0.90)** |
| **0.61 (0.55–0.67)** | **0.69 (0.59–0.76)** | **1.43 (1.26–1.64)** | Ref (1.00) | **1.13 (1.01–1.26)** |
| **0.54 (0.49–0.60)** | **0.60 (0.52–0.69)** | **1.27 (1.11–1.47)** | **0.89 (0.79–0.99)** | Ref (1.00) |
| | | **Cancer** | | |
| Ref (1.00) | 0.94 (0.86–1.03) | 0.97 (0.87–1.09) | 1.05 (0.97–1.13) | **1.15 (1.04–1.26)** |
| 1.06 (0.97–1.16) | Ref (1.00) | 1.04 (0.91–1.18) | **1.11 (1.00–1.24)** | **1.22 (1.08–1.37)** |
| 1.03 (0.92–1.15) | 0.97 (0.85–1.01) | Ref (1.00) | 1.08 (0.95–1.22) | **1.18 (1.03–1.35)** |
| 0.95 (0.88–1.03) | **0.90 (0.81–1.00)** | 0.93 (0.82–1.05) | Ref (1.00) | 1.09 (0.98–1.22) |
| **0.87 (0.79–0.96)** | **0.82 (0.73–0.93)** | **0.85 (0.74–0.97)** | 0.91 (0.82–1.02) | Ref (1.00) |
| | | **CRDs** | | |
| Ref (1.00) | **0.86 (0.78–0.95)** | **1.28 (1.14–1.44)** | **1.20 (1.11–1.30)** | **1.26 (1.14–1.40)** |
| **1.16 (1.05–1.29)** | Ref (1.00) | **1.49 (1.30–1.70)** | **1.40 (1.25–1.56)** | **1.47 (1.29–1.67)** |
| **0.78 (0.70–0.88)** | **0.67 (0.59–0.77)** | Ref (1.00) | 0.94 (0.83–1.06) | 0.99 (0.86–1.13) |
| **0.83 (0.77–0.90)** | **0.72 (0.64–0.80)** | 1.06 (0.94–1.20) | Ref (1.00) | 1.05 (0.94–1.17) |
| **0.79 (0.71–0.88)** | **0.68 (0.60–0.77)** | 1.01 (0.88–1.16) | 0.95 (0.86–1.06) | Ref (1.00) |
| | | **CVDs** | | |
| Ref (1.00) | 0.89 (0.79–1.02) | **1.27 (1.09–1.48)** | **1.15 (1.05–1.27)** | 1.10 (0.97–1.24) |
| 1.12 (0.98 –1.27) | Ref (1.00) | **1.42 (1.19–1.70)** | **1.29 (1.12–1.48)** | **1.23 (1.05–1.44)** |
| **0.79 (0.68–0.92)** | **0.70 (0.59–0.84)** | Ref (1.00) | 0.91 (0.77–1.07) | 0.86 (0.72–1.03) |
| **0.87 (0.79–0.96)** | **0.78 (0.67–0.89)** | 1.10 (0.94–1.30) | Ref (1.00) | 0.95 (0.84–1.08) |
| 0.91 (0.81–1.03) | **0.82 (0.70–0.96)** | 1.16 (0.97–1.38) | 1.05 (0.92–1.20) | Ref (1.00) |

Bold means results have statistical meanings, $p < 0.05$; Red means the LPs play as risk factors for disease risk, and blue represents protective effects

**Table S3** AIC values for non-linear models of lifestyle summation scores and disease risk, with degrees of freedom ranging from 2 to 6

| | | Degree of freedom | AIC | Optimal model |
|---|---|---|---|---|
| **Healthy lifestyle scores** | | | | |
| T2D | Non-linear model 1 | 2 | 65036.23 | |
| | Non-linear model 2 | 3 | 65037.52 | |
| | Non-linear model 3 | 4 | 65039.32 | df=2 |
| | Non-linear model 4 | 5 | 65041.21 | |
| | Non-linear model 5 | 6 | 65041.54 | |
| Cancer | Non-linear model 1 | 2 | 98763.03 | |
| | Non-linear model 2 | 3 | 98764.70 | |
| | Non-linear model 3 | 4 | 98766.70 | df=2 |
| | Non-linear model 4 | 5 | 98768.46 | |
| | Non-linear model 5 | 6 | 98769.61 | |
| CRDs | Non-linear model 1 | 2 | 88229.71 | |
| | Non-linear model 2 | 3 | 88231.15 | |
| | Non-linear model 3 | 4 | 88229.26 | df=4 |
| | Non-linear model 4 | 5 | 88230.88 | |
| | Non-linear model 5 | 6 | 88232.88 | |
| CVDs | Non-linear model 1 | 2 | 57376.92 | |
| | Non-linear model 2 | 3 | 57378.46 | |
| | Non-linear model 3 | 4 | 57376.61 | df=4 |
| | Non-linear model 4 | 5 | 57378.50 | |
| | Non-linear model 5 | 6 | 57380.43 | |
| **Unhealthy lifestyle scores** | | | | |
| T2D | Non-linear model 1 | 2 | 64957.35 | |
| | Non-linear model 2 | 3 | 64959.28 | |
| | Non-linear model 3 | 4 | 64956.76 | df=4 |
| | Non-linear model 4 | 5 | 64957.53 | |
| | Non-linear model 5 | 6 | 64959.11 | |
| Cancer | Non-linear model 1 | 2 | 98788.89 | |
| | Non-linear model 2 | 3 | 98788.64 | |
| | Non-linear model 3 | 4 | 98789.98 | df=3 |
| | Non-linear model 4 | 5 | 98790.76 | |
| | Non-linear model 5 | 6 | 98792.14 | |
| CRDs | Non-linear model 1 | 2 | 88226.20 | |
| | Non-linear model 2 | 3 | 88226.11 | |
| | Non-linear model 3 | 4 | 88228.12 | df=3 |
| | Non-linear model 4 | 5 | 88229.84 | |
| | Non-linear model 5 | 6 | 88231.27 | |
| CVDs | Non-linear model 1 | 2 | 57373.72 | |
| | Non-linear model 2 | 3 | 57375.12 | |
| | Non-linear model 3 | 4 | 57376.41 | df=2 |
| | Non-linear model 4 | 5 | 57376.98 | |
| | Non-linear model 5 | 6 | 57378.98 | |

AIC: Akaike information criterion; *df*: degree of freedom

**Table S4** Associations between LPs and disease risk

| | T2D incidence | Cancer incidence | CRDs incidence | CVDs incidence |
|---|---|---|---|---|
| **LPs** | | | | |
| Unhealthy | 1(ref) | 1(ref) | 1(ref) | 1(ref) |
| Healthy in a balanced way | **0.55 (0.49–0.61)** | **0.87 (0.79–0.96)** | **0.80 (0.72–0.89)** | 0.92 (0.82–1.04) |
| Healthy but physically inactive | **0.60 (0.52–0.69)** | **0.82 (0.73–0.93)** | **0.68 (0.60–0.78)** | **0.82 (0.70–0.96)** |
| Unhealthy but no substance use | **1.28 (1.11–1.48)** | **0.85 (0.74–0.97)** | 1.01 (0.88–1.16) | 1.16 (0.97–1.38) |
| Unhealthy but light drinking and never smoked | 0.89 (0.80–1.00) | 0.92 (0.82–1.02) | 0.96 (0.86–1.07) | 1.06 (0.93–1.21) |

All models adjusted for age, sex, education attainment, marital status, income, other diseases' status and employment status

It reports sub–hazard ratios (SHR) (95% confidence interval)

Bold means the SHR met statistical significance: $p < 0.05$

**Table S5** Associations between LPs and disease risk, complete-case analysis

| | T2D incidence | Cancer incidence | CRDs incidence | CVDs incidence |
|---|---|---|---|---|
| **Sample size** | **85,320** | **96,750** | **68,105** | **57,576** |
| **LPs** | | | | |
| Unhealthy | 1(ref) | 1(ref) | 1(ref) | 1(ref) |
| Healthy in a balanced way | **0.58 (0.51–0.66)** | 0.96 (0.86–1.07) | **0.84 (0.75–0.95)** | 0.99 (0.86–1.15) |
| Healthy but physically inactive | 0.85 (0.71–1.02) | 0.91 (0.78–1.07) | 1.01 (0.86–1.18) | 1.05 (0.85–1.29) |
| Unhealthy but no substance use | **1.38 (1.16–1.62)** | 1.01 (0.85–1.19) | 1.09 (0.93–1.28) | **1.36 (1.10–1.68)** |
| Unhealthy but light drinking and never smoked | **0.86 (0.76–0.98)** | 0.94 (0.83–1.06) | 0.92 (0.82–1.04) | 1.08 (0.93–1.26) |

All models adjusted for age, sex, education attainment, marital status, income and employment status

It reports sub-hazard ratios (SHR) (95% confidence interval)

Bold means the SHR met statistical significance: $p < 0.05$

**Table S6** Associations between LPs and disease risk

| | T2D incidence | Cancer incidence | CRDs incidence | CVDs incidence |
|---|---|---|---|---|
| **Lifestyle patterns** | | | | |
| Unhealthy | 1(ref) | 1(ref) | 1(ref) | 1(ref) |
| Healthy in a balanced way | **0.53 (0.49–0.65)** | **0.97 (0.95–0.97)** | **0.78 (0.70–0.86)** | 0.92 (0.82–1.04) |
| Healthy but physically inactive | **0.57 (0.49–0.65)** | **0.96 (0.95–0.97)** | **0.67 (0.59–0.76)** | **0.85 (0.72–0.99)** |
| Unhealthy but no substance use | **1.32 (1.15–1.52)** | **0.97 (0.95–0.98)** | 1.03 (0.90–1.18) | **1.19 (1.00–1.42)** |
| Unhealthy but light drinking and never smoked | 0.91 (0.81–1.01) | 0.99 (0.98–1.00) | 0.95 (0.86–1.06) | 1.02 (0.90–1.16) |

All models adjusted for age, sex, education attainment, marital status, income and employment status

It reports hazard ratios (HR) (95% confidence interval) with cox proportional hazard models

Bold means the SHR met statistical significance: $p$ <0.05

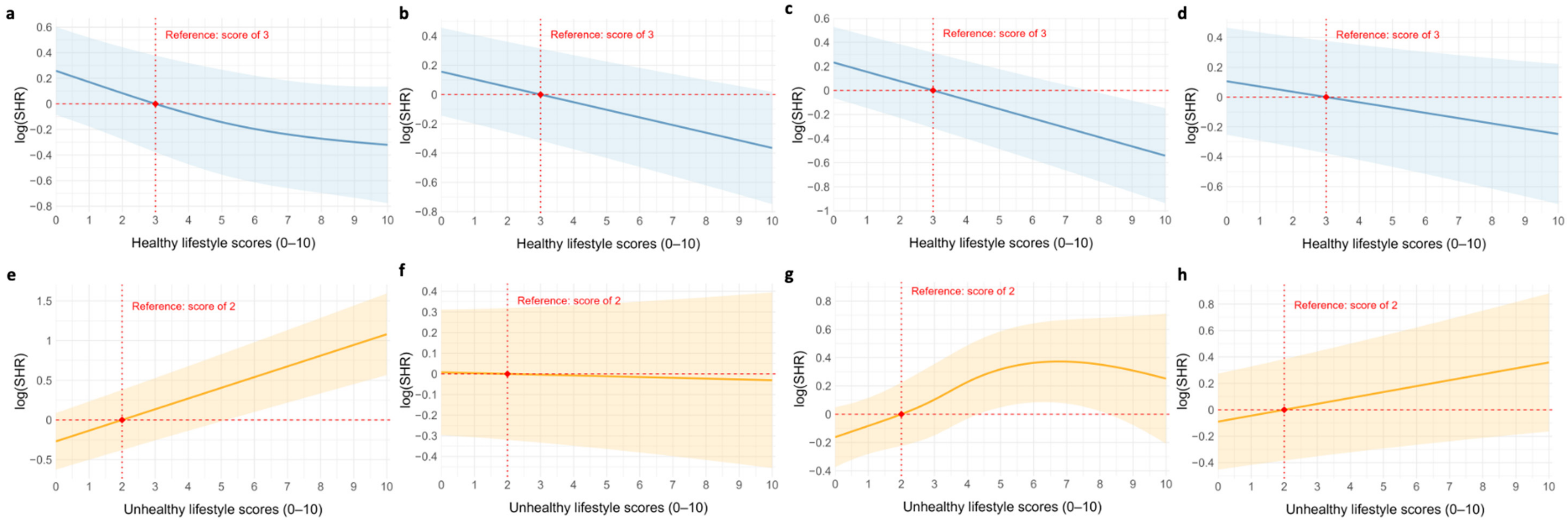


**Figure S2** Association curves between lifestyle summation scores and disease risk

Unhealthy lifestyle score was treated as a covariate when analysing associations between the healthy lifestyle score and NCD incidence, and vice versa. Light blue and light orange zones showed corresponding 95% confidence intervals. a&e, risk for type 2 diseases; b&f risk for cancer; c&g risk for respiratory diseases; and d&h risk for cardiovascular diseases. All models adjusted for age (median), sex (female), marital status (married/cohabiting), income (1500–1900), highest education (upper secondary) and employment status (full-time employed).

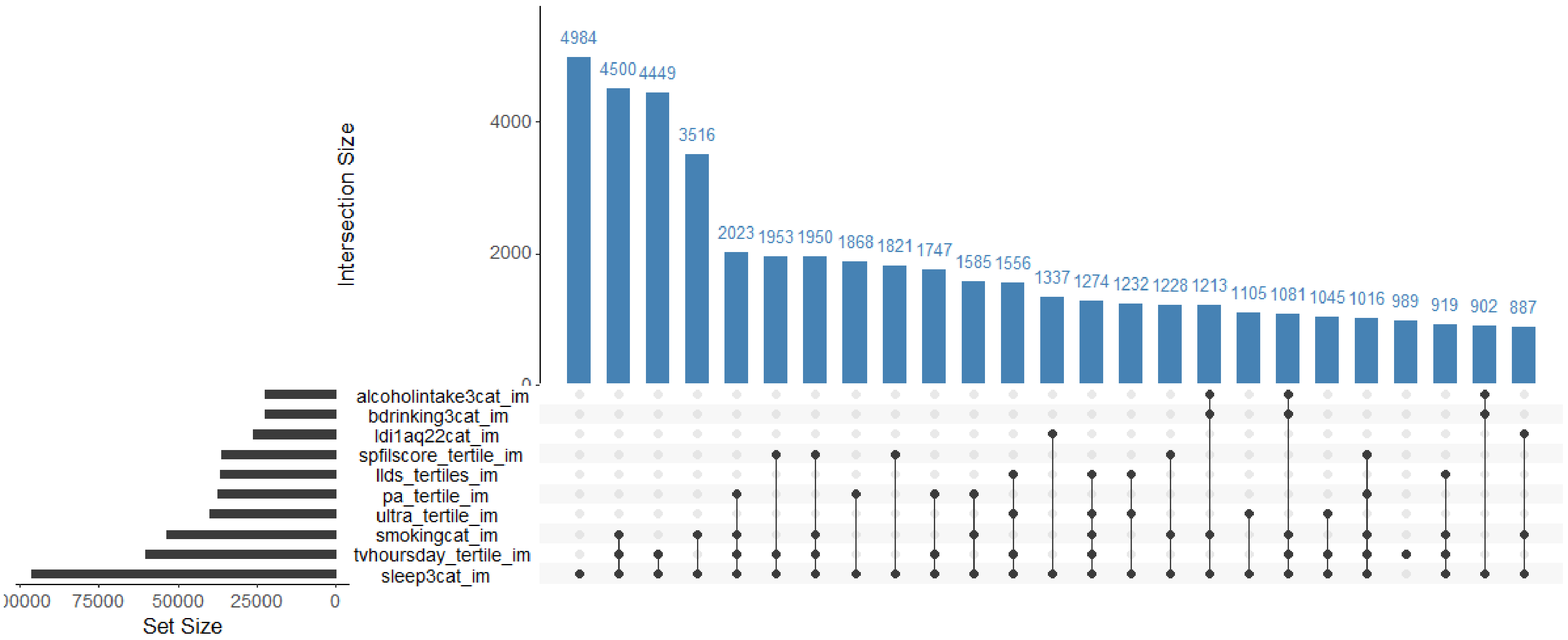


**Figure S3** Upset plot illustrating the prevalence of different combinations of healthy lifestyle factors

It shows the top 25% most prevalent combinations of multiple lifestyle factors. From top to bottom, the lifestyle factors are: alcohol intake (abstainer), binge drinking (abstainer), stress levels (low), social connection (high), diet quality (high), physical activity (high), ultra-processed food consumption (low), smoking status (never smoking), TV watching (<= 2 hours/day), and sleeping habits (recommended).

**Table S7** Model Comparison with null models for LPs

| | T2D model | Cancer model | CRDs model | CVDs model |
|---|---|---|---|---|
| **Null log-likelihood (β = 0)** | -33258.91 | -50330.48 | -44281.07 | -29700.84 |
| **Full log-likelihood** | -32496.46 | -49370.41 | -44142.58 | -28668.19 |

Higher log-likelihood represents better model fit.

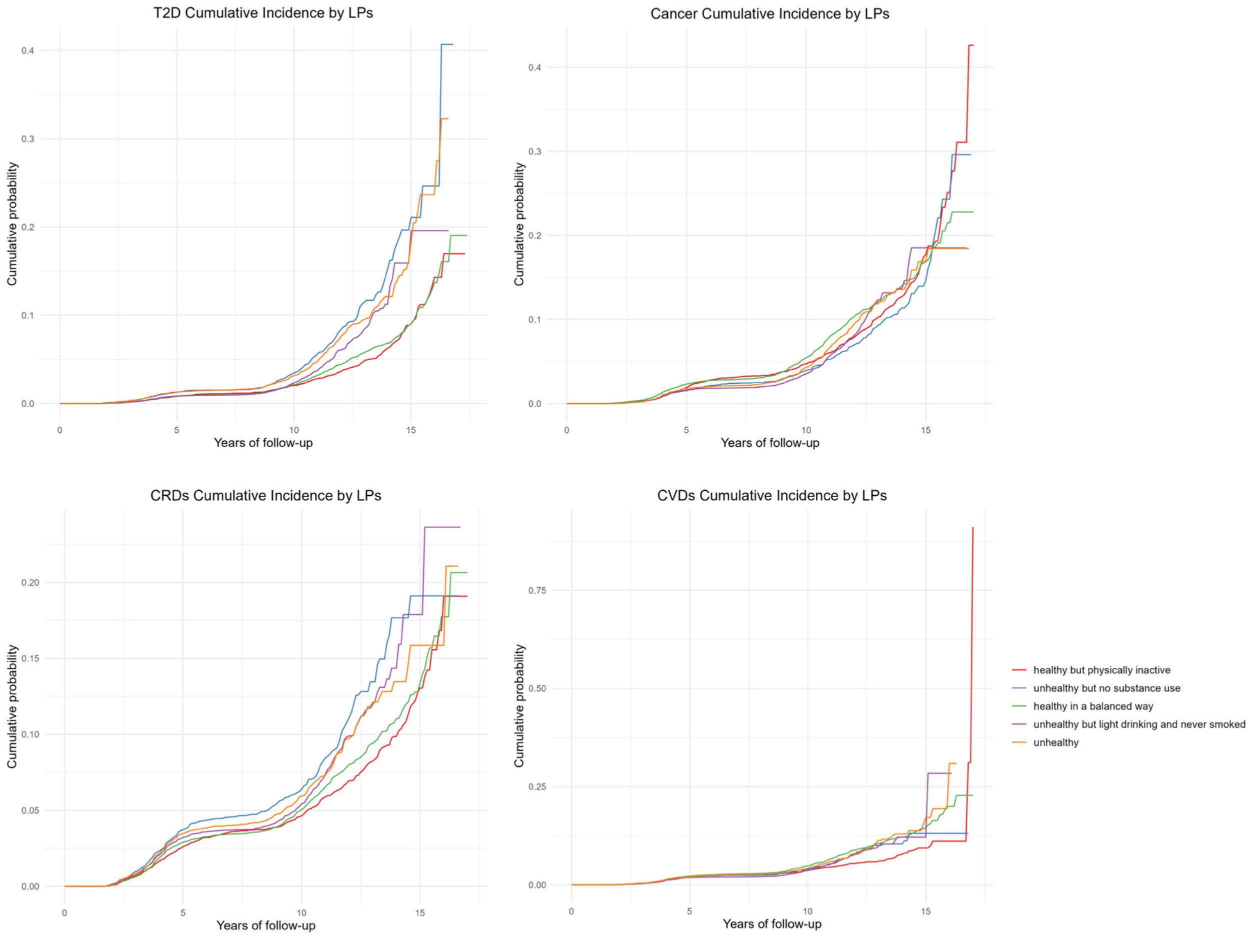


**Figure S4** Cumulative incidence curves of diseases by LPs
