# Supplementary material for: Lifestyle Patterns and Incidence of Cardiovascular Diseases, Cancer, Respiratory Diseases, and Type 2 Diabetes: A Large-Scale Prospective Cohort Study
Source: Nutrients. 2025 Dec 12;17(24):3883. doi: 10.3390/nu17243883 (PMC12736167; doi:10.3390/nu17243883)
Supplement: Supplementary file 1 [file nutrients-17-03883-s001.zip › Supplementary S1.pdf]

Supplementary Material File S1: Definitions of confounders

Confounders

Marital status was categorised into four groups: (1) married or cohabiting, (2) in a relationship but not cohabiting, (3) no partner (including widowed individuals), and (4) others. Household income was normalized by dividing net household income (the midpoint of each participant's income category) by the square root of the number of household members. Based on this normalized income, six categories were created: (1) less than €1100, (2) €1100 to €1500, (3) €1500 to €1900, (4) more than €1900, (5) "I don't know this", and (6) "I don't want to tell". Educational attainment was classified into five levels: (1) elementary education (no education or primary education), (2) lower secondary education (lower or preparatory vocational education or lower general secondary education), (3) upper secondary education (intermediate vocational education, apprenticeship, higher general secondary education, or pre-university secondary education), (4) tertiary education (higher vocational education or university), and (5) others. Employment status was categorized into seven groups: (1) full-time employment, (2) retired, (3) housewife/husband, (4) student, (5) not employed (including unemployed individuals, job seekers, those unfit for work, or receiving social assistance benefits), (6) part-time employment (12 to 32 hours per week), and (7) employment for fewer than 12 hours per week.
